# Phylogeny and Historical Biogeography of the Scorpion Genus *Hottentotta* Birula, 1908 (Buthidae) in the Iranian Plateau and the Zagros Mountains

**DOI:** 10.3390/insects17030239

**Published:** 2026-02-25

**Authors:** Omid Mirshamsi, Masoumeh Amiri, Mansour Aliabadian, Lorenzo Prendini

**Affiliations:** 1 Department of Biology, Faculty of Science, Ferdowsi University of Mashhad, Mashhad 9177948974, Iran; masoumeh.amiri@um.ac.ir (M.A.); aliabadi@um.ac.ir (M.A.); 2 Research Department of Zoological Innovations, Institute of Applied Zoology, Faculty of Science, Ferdowsi University of Mashhad, Mashhad 9177948974, Iran; 3 Arachnology Lab and Scorpion Systematics Research Group, Division of Invertebrate Zoology, American Museum of Natural History, New York, NY 10024, USA

**Keywords:** paleobiogeography, Iranian Plateau, Zagros Mountains, dispersal, vicariance

## Abstract

The scorpion genus *Hottentotta* Birula, 1908 is widely distributed across Africa and the Middle East, extending to Pakistan, India and Sri Lanka. The processes which resulted in their evolution and diversification across this vast area are poorly understood. The present study investigated the phylogeny and historical biogeography of the genus in the Iranian Plateau and the Zagros Mountains based on nuclear and mitochondrial DNA sequences from four African species, an Arabian species and eight species from the Middle East, most of which are endemic to Iran. Phylogenetic analyses confirmed the monophyly of all species included in the analysis and recovered a clade comprising Iranian and Afro-Arabian species. S-DIVA and BBM analyses demonstrated that the species of *Hottentotta* occurring in the Iranian Plateau and the Zagros Mountains originated from an African ancestor and then dispersed to their current geographical ranges. Further divergence coincided with the orogeny of the Zagros Mountains and climatic changes during the Miocene epoch. The results support the hypothesis that the Zagros Mountains formed a geographical barrier which promoted vicariance and diversification on the Iranian Plateau.

## Simple Summary

The scorpion genus *Hottentotta* is widely distributed across Africa, the Middle East, and parts of South Asia, but its evolutionary history is not well understood. In this study, we analyzed nuclear and mitochondrial DNA from species found in Africa, Arabia, and the Middle East, with a focus on species from Iran. Our results confirmed that all examined species form a single evolutionary group and that Iranian and Afro-Arabian species share a common ancestor. Biogeographic analyses suggest that *Hottentotta* species in the Iranian Plateau and Zagros Mountains originated from an African ancestor that later dispersed into the region. Their diversification appears to have been influenced by the uplift of the Zagros Mountains and climate changes during the Miocene epoch. These findings support the idea that the Zagros Mountains acted as a geographic barrier, promoting species separation and diversification in the Iranian Plateau.

## 1. Introduction

Geographical barriers play a prominent role in hindering gene flow, which can lead to genetic differentiation among allopatric populations and, ultimately, speciation over evolutionary time scales [1,2,3,4,5]. Numerous investigations have underscored the roles of the Iranian Plateau and the Zagros Mountains, which harbor one of the most diverse biotas in southwestern Asia [6,7,8,9,10], as drivers of diversification in the Middle East [11,12,13,14,15,16,17,18,19]. The Zagros Mountains extend from southeastern Turkey, along the western margin of the Iranian Plateau, to the Makran subduction zone [20]. Formed by the collision of the Arabian and Eurasian tectonic plates during the Late Miocene to Early Pliocene, approximately 11.63–3.6 million years ago (Ma) [21], the orogeny of the Zagros Mountains, along with the emergence of the Kavir and Lut deserts, greatly influenced the evolution of the fauna on the Iranian Plateau [22].

Several studies have emphasized the significance of scorpions as model organisms for understanding the roles of geomorphology and paleoclimatic shifts on diversification [18,23,24,25,26,27,28,29]. The Iranian Plateau and the Zagros Mountains represent a biodiversity hotspot for scorpions, with over 80 species documented to date [30,31]. The mostly arid to semi-arid environment of this region created many favorable habitats for scorpions, particularly the diverse family Buthidae C.L. Koch, 1837, which contains several speciose genera distributed throughout the Palearctic deserts. One such genus, *Hottentotta* Birula, 1908, comprising approximately 60 species, occurs across the savannas and semi-deserts of Africa and the Middle East, from the Arabian Peninsula to Pakistan, India and Sri Lanka [12,30,32].

Twelve species of *Hottentotta* are currently recorded from Iran [12,32,33,34,35]. The distribution of *Hottentotta* across the Zagros Mountains and the Iranian Plateau renders these scorpions particularly interesting for investigating the processes that shaped arthropod diversification across the region. The present investigation estimated the phylogeny of *Hottentotta* in the Iranian Plateau and the Zagros Mountains using a multilocus dataset for an extensive taxon sample, including four African species, one Arabian species, and eight endemic species from Iran. A time-calibrated tree was generated to reconstruct the biogeographical history of *Hottentotta* in the Iranian Plateau and the Zagros Mountains and illuminate the geomorphological processes that influenced their diversification across the region.

## 2. Materials and Methods

### 2.1. Taxon Sampling

Nine of the twelve species of *Hottentotta* recorded from Iran were included in the present investigation: *Hottentotta hatamtiorum* Amiri et al., 2024; *Hottentotta jayakari* (Pocock, 1895); *Hottentotta juliae* Kovařík et al., 2019; *Hottentotta khoozestanus* Navidpour et al., 2008; *Hottentotta navidpouri* Kovařík et al., 2018; *Hottentotta saulcyi* (Simon, 1880); *Hottentotta schach* (Birula, 1905); *Hottentotta sistanensis* Kovařík et al., 2018; and *Hottentotta zagrosensis* Kovařík, 1997. All of these species are endemic to Iran except for *H. saulcyi*, which also occurs in Iraq and southeastern Turkey, and *H. jayakari*, which is endemic to the Arabian Peninsula and several islands in the Persian Gulf, which form part of the territory of Iran. The three remaining Iranian species, i.e., *Hottentotta akbarii* Yağmur et al., 2022, *Hottentotta lorestanus* Navidpour et al., 2010, and *Hottentotta pooyani* Moradi et al., 2022, each described from only a single specimen, are of dubious taxonomic validity.

Four African species of *Hottenotta*, i.e., the type species, *Hottentotta hottentotta* (Fabricius, 1787), from west Africa, *Hottentotta franzwerneri* (Birula, 1914) and *Hottentotta gentili* (Pallary, 1924), from the Maghreb, and *Hottentotta minax* (L. Koch, 1875), from east Africa, were also included in the ingroup. Two Palearctic buthid taxa, i.e., *Androctonus crassicauda* (Olivier, 1807) and *Mesobuthus eupeus* (C.L. Koch, 1839), served as outgroups and the tree was rooted on *A. crassicauda.* Where all sequences of the species occurring in Iran were newly generated for the study, sequences of the African species (one mitochondrial gene only) were obtained from GenBank (Table 1).

### 2.2. Material and Mapping

Specimens were collected using ultraviolet light detection at night and rock-rolling during daytime. Freshly collected material, transferred to 75–96% ethyl alcohol, was deposited at the American Museum of Natural History (AMNH), New York, NY, USA, and the Zoological Museum at Ferdowsi University of Mashhad (ZMFUM), Iran. Tissue samples were archived in the Ambrose Monell Cryocollection (AMCC) at the AMNH.

**Figure 1:**
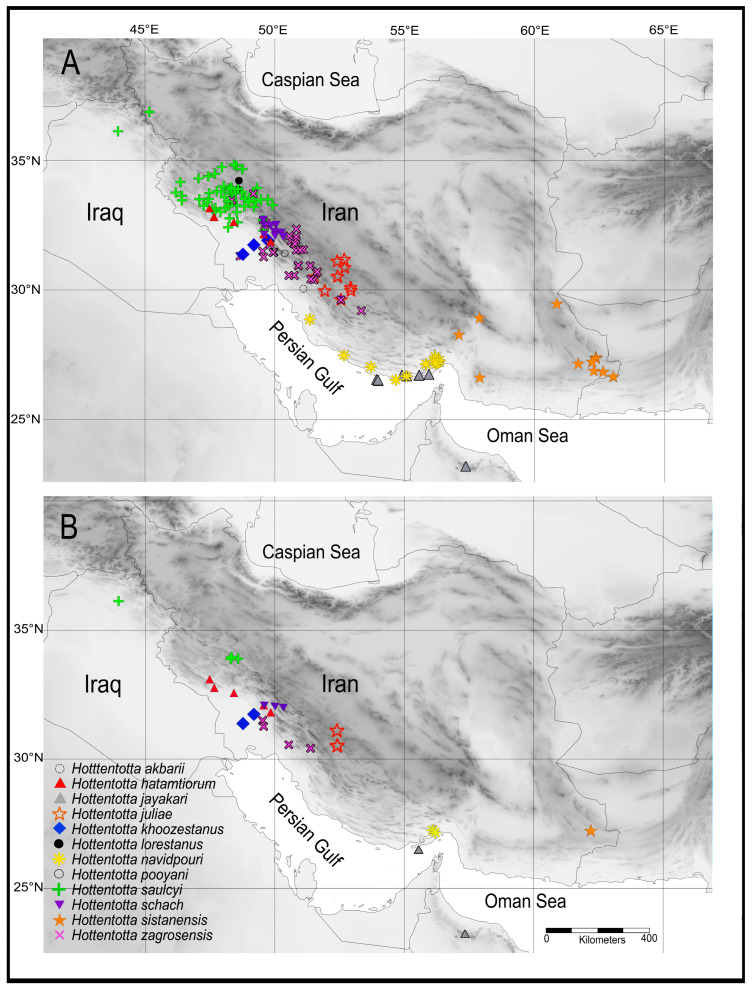
Map plotting locality records of *Hottentotta* Birula, 1908 recorded from Iran (**A**) and material from which DNA was extracted and sequenced for the present investigation (**B**).

A distribution map (Figure 1) was created using DIVA-GIS [36] by overlaying point locality records of sampling locations on spatial layers depicting political boundaries and topography (elevation) at 2.5 arc-minutes altitude.

### 2.3. DNA Extraction, Amplification, Sequencing and Alignment

DNA was extracted from leg muscle tissue using the Favorgene (Taipei, Taiwan) DNA Extraction kit or the Qiagen (Hilden, Germany) DNeasy Blood and Tissue Kit. DNA sequences were generated from one nuclear gene locus, 28S rDNA (hereafter, 28S), and three mitochondrial gene loci, 12S rDNA (hereafter, 12S), 16S rDNA (hereafter, 16S) and Cytochrome *c* Oxidase Subunit I (hereafter, COI), amplified using standard primers [28,37,38]. The polymerase chain reaction (PCR) was performed with a variety of optimization protocols [13,28,37,38,39]. Each PCR contained 12.5 μL Ampliqon (Odense, Denmark) ready master mix, 7.5 μL deionized H_2_O, 1 μL (=1 pmol) of each primer and 3 μL of DNA template. PCR products were Sanger dideoxy sequenced using ABI Big Dye terminator chemistry on an ABI Prism 3700 (Applied Biosystems, Foster City, CA, USA). A total of 78 sequences were newly generated from 32 samples for this study and deposited in GenBank (Table 1).

Sequences were edited and assembled using Sequencher v.4.5.6 (GeneCodes Corporation, Ann Arbor, MI, USA). Edited sequences of the four gene loci were aligned using MUSCLE [40]. The protein coding locus, COI, was translated into amino acids to evaluate its quality and the coding frame adjusted by identifying stop codons MEGA X [41].

### 2.4. Phylogenetic Analysis

In the 513 base pair (bp) alignment of 28S, 506 (98.6%) positions were conserved, seven (1.4%) were variable, and six (1.2%) were parsimony informative. In the 343 bp alignment of 12S, 206 (60%) positions were conserved, 137 (40%) were variable, and 110 (32%) were parsimony informative. In the 496 bp alignment of 16S, 324 (65%) positions were conserved, 170 (35%) were variable, and 146 (29.4%) were parsimony informative. In the 657 bp alignment of COI, 450 (68.50%) positions were conserved, 207 (31.50%) were variable, and 181 (27.55%) were parsimony informative. The best-fitting models of nucleotide substitution were estimated for each gene locus using the Akaike Information Criterion [42] in jModeltest v.2.1.10 [43], and a tree estimated from the concatenated alignment of 2009 bp using GTR + I + G.

Analysis of the concatenated dataset with Bayesian Inference (BI) was conducted using MrBayes v.3.2 [44], applying the best-fit models to each gene locus. The analysis was performed using two independent runs with six chains for 1.5 × 10^7^ generations. Subsampling trees and parameters were saved every 1000th generation. Convergence on the stationary distribution was assessed with Tracer v.1.7.2 [45]. Independent runs converged well (ESS > 400 for all parameters; ASDSF = 0.0056), indicating adequate sampling of the posterior. Bayesian tree and posterior probabilities were calculated by 50% majority-rule consensus after burning off all pre-asymptotic topologies.

Kimura 2-parameter (K2P) pairwise genetic distances were calculated among and within species for each gene locus using MEGA X [41] (Table 2).

### 2.5. Divergence Time Estimation

Divergence time estimation was calculated with the GTR + G + I model applied to each gene locus in the concatenated dataset, using BEAST v.1.10.4 [46]. Site and clock models were unlinked across partitions. A relaxed clock (uncorrected) with lognormal prior distribution was applied with a Yule model process as tree prior. The *ucld.mean* of the clock model was set to COI and 16S scorpion-specific mutation rates of 0.007 and 0.005 substitution/site/myr, respectively [47,48]. The analysis was run for 5 × 10^7^ generations, sampling every 1000th generation. Convergence diagnostics for the MCMC analyses were checked in Tracer v.1.7.2 [49] to ensure that all ESS values were greater than 200. A lineage-through-time (LTT) analysis was conducted on the concatenated dataset using Tracer v.1.7.2.

### 2.6. Biogeographical Analysis

Statistical dispersal-vicariance (S-DIVA) analysis [50] was conducted using RASP v.4.4 [51] in BioGeoBears [52] to reconstruct ancestral ranges. The maximum clade credibility tree topology was input from BEAST with 50,000 topologies used to account for phylogenetic uncertainty. A statistical comparison of six models (DEC, DEC + J, DIVALIKE, DIVALIKE + J, BAYAREALIKE and BAYAREALIKE + J), performed using the Likelihood Ratio test (LRT), suggested BAYAREALIKE + J as the best fitting model for estimating ancestral ranges (AICc_wt = 0.41; Table 3). Species distributions were assigned to five geographical regions: (A) northern Zagros fold; (B) southern Zagros fold; (C) Makran; (D) Arabian Peninsula; (E) Africa. Analyses were conducted with dispersal only possible between adjoining areas, allowing for extinction, and only two-unit areas in the ancestral distributions.

Bayesian binary Markov Chain Monte Carlo (MCMC) (BBM) analysis [50,51] was also performed with eight MCMC chains running simultaneously for 5 × 10^6^ generations, and states sampled every 100 generations. A fixed Jukes-Cantor model with equal among-site rate variation was applied for the analysis.

## 3. Results

### 3.1. Phylogenetic Analysis

**Figure 2:**
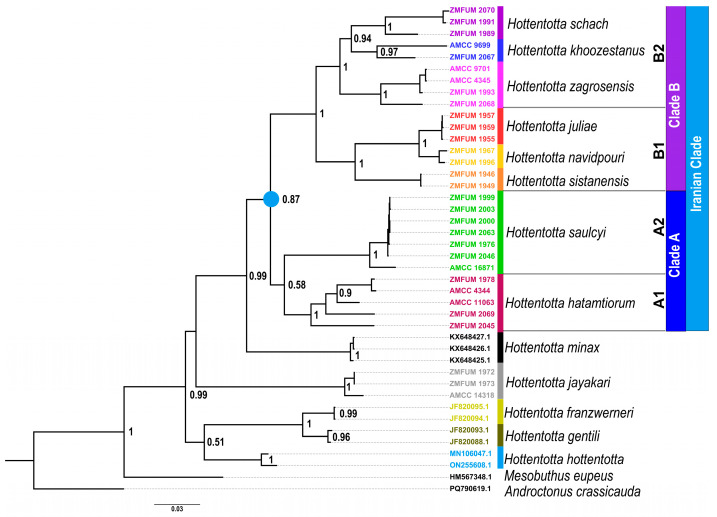
Bayesian Inference (BI) phylogeny of *Hottentotta* Birula, 1908, based on 2009 aligned nucleotide base pairs of nuclear and mitochondrial DNA. Nodal support values above branches represent posterior probability values from BI and bootstrap values from Maximum Likelihood analyses. Scale bar represents number of substitutions per nucleotide site. Terminal colors correspond to Figure 1. The blue circle denotes the Iranian clade.

The tree topology obtained with BI confirmed the monophyly of *Hottentotta* (posterior probability, PP = 0.99) and all *Hottentotta* species included in the analysis (PP = 0.96–1) and recovered a well-supported clade comprising the species from the Iranian Plateau and the Zagros Mountains (PP = 0.87), in turn divided into two well-supported clades (Figure 2): Clade A, comprising *H. hatamtiorum* (Clade A_1_) and *H. saulcyi* (Clade A_2_), and Clade B, comprising the other six species. Clade B was further subdivided into two clades, Clade B_1_ comprising *H. sistanensis*, placed sister to a subclade comprising *H. juliae* and *H. navidpouri*, and Clade B_2_ comprising *H. zagrosensis*, placed sister to a subclade comprising *H. khoozestanus* and *H. schach*.

The African species of *Hottentotta* were paraphyletic with respect to the Asian species. *Hottentotta minax*, from east Africa, was placed sister to the clade of Iranian species, and *H. jayakari*, from the Arabian Peninsula and islands in the Persian Gulf, was placed sister to the clade comprising *H. minax* and the Iranian clade. A weakly supported clade (PP = 0.51) comprising *H. hottentotta* from west Africa and the two Maghreb species, *H. franzwerneri* and *H. gentili*, formed the basal sister group of all other species.

### 3.2. Genetic Distances

The average genetic distance between the species of *Hottentotta* varied from 5.2 to 22% for 12S, 6.7–23.7% for 16S, 5.0–17% for COI, and 0.1–0.7% for 28S (Table 2). The highest interspecific distance was observed between *H. jayakari* and the other species. The lowest interspecific distances for the mitochondrial loci were observed between *H. juliae* and *H. navidpouri*, and between *H. franzwerneri* and *H. gentili*. Intraspecific genetic distances varied from 0.0 to 9.0% for 12S, 0.0–5.0%, for 16S and 0.0–8.0% for COI.

### 3.3. Divergence Time Estimation

**Figure 3:**
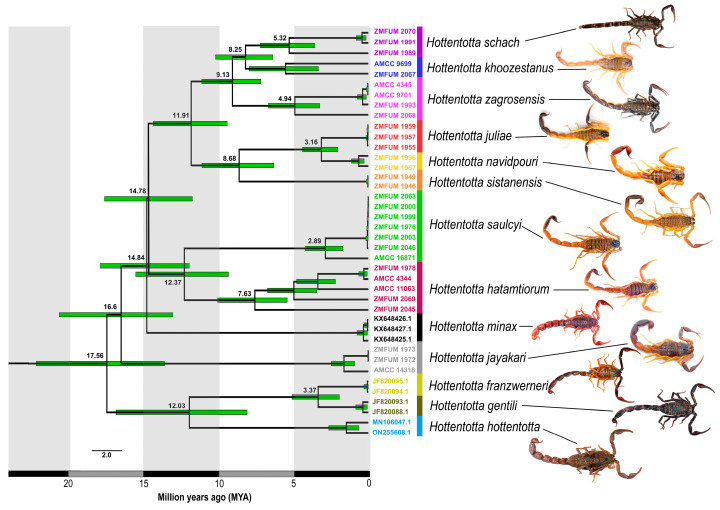
Time-calibrated phylogeny of *Hottentotta* Birula, 1908, based on 2009 aligned nucleotide base pairs of nuclear and mitochondrial DNA. Green bars indicate 95% highest posterior density intervals for estimated node ages; mean node ages (Ma) are provided below branches. Terminal colors correspond to Figure 1.

According to the divergence time estimations (Figure 3), the common ancestor of the west African *H. hottentotta* and the Maghreb species, *H. franzwerneri* and *H. gentili*, diverged from the common ancestor of all other species (Clades A, B, *H. jayakari* and *H. minax*) around 17.56 Ma (95% highest posterior density, HPD: 13.68–22.31), whereas the Arabian *H. jayakari* and the east African *H. minax* diverged from the common ancestor of Clades A and B 16.6 Ma (95% HPD: 13.12–20.76) and 14.84 Ma (95% HPD: 12.03–18), respectively. Clade A, comprising *H. hatamtiorum* and *H. saulcyi*, diverged from Clade B, comprising the other Iranian species, around 14.78 Ma (95% HPD: 11.82–17.72). *Hottentotta hatamtiorum* diverged from *H. saulcyi* around 12.37 Ma (95% HPD: 9.4–15.62). Clades B_1_ and B_2_ diverged nearly 11.91 Ma (95% HPD: 9.48–14.43). In Clade B_1_, *H. sistanensis* diverged from the common ancestor of *H. juliae* and *H. navidpouri* around 8.68 Ma (95% HPD: 6.35–11.19), whereas the latter species diverged around 3.16 Ma (95% HPD: 2.05–4.43). In Clade B_2_, *H. zagrosensis* diverged from the common ancestor of *H. khoozestanus* and *H. schach* around 9.13 Ma (95% HPD: 7.22–11.2), whereas the latter species diverged around 8.25 Ma (95% HPD: 6.42–10.27).

### 3.4. Biogeographical Analysis

**Figure 4:**
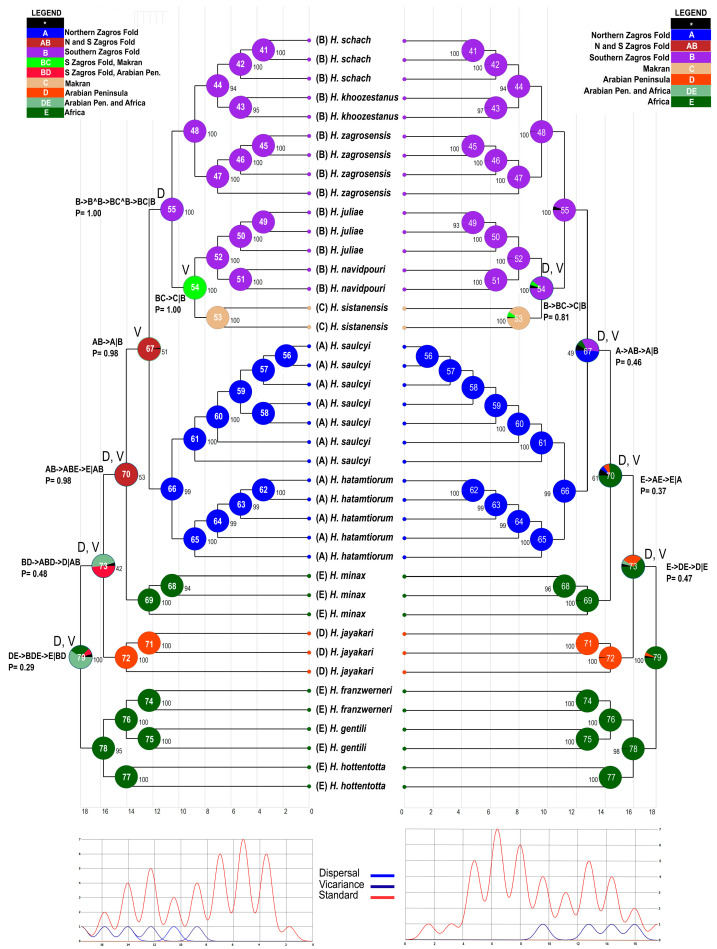
Biogeographical analyses of *Hottentotta* Birula, 1908, based on 2009 aligned nucleotide base pairs of nuclear and mitochondrial DNA, using S-DIVA (**left**) and BBM (**right**). The five regions are as follows: northern Zagros fold; southern Zagros fold; Makran region; Arabian Peninsula; India. Green (V) and blue (D) circles around nodes indicate vicariance and dispersal events, respectively. Color key and letters A–E indicate ancestral ranges; letter combinations indicate a combination of ranges; and black with an asterisk represents other ancestral ranges. Event route and probability are provided for nodes 54, 55, 67, 70, 73 and 79.

The results of S-DIVA and BBM were similar, revealing dispersal and vicariance events at the main ancestral nodes (Figure 4; Table 3). The ancestral node (79) of *Hottentotta* underwent one dispersal event and one vicariance event, which reflects range expansion followed by geographical partitioning, according to S-DIVA. The ancestral node (73) of *H. jayakari*, *H. minax*, and the Iranian clade underwent one dispersal event and one vicariance event according to both S-DIVA and BBM (relative probabilities of 0.48 and 0.47, respectively). These events led to divergence of the Arabian *H. jayakari* from the common ancestor of the east African *H. minax* and the Iranian species. The ancestral node (70) of the clade comprising *H. minax* and the Iranian species underwent one dispersal event and one vicariance event according to both S-DIVA and BBM, resulting in the divergence of *H. minax* from the common ancestor of the Iranian clade.

These events suggest recolonization of Africa via a single dispersal event, followed by vicariance which partitioned the ancestral range between areas E and A + B (relative probability = 0.98). The ancestral node (67) of the Iranian species underwent one vicariance event according to S-DIVA compared with one dispersal event and one vicariance event according to BBM. These events caused the divergence of the species occurring in the northern Zagros Mountains from those occurring in the southern Zagros Mountains and the Makran region (S-DIVA; relative probability: 0.98).

According to the S-DIVA analysis, four dispersal events and five vicariance events occurred among the species of *Hottentotta*. One of these is a vicariance event separating the species of the northern Zagros Mountains from those of the southern Zagros Mountains and the Makran region (node 67; relative probability: 0.98). Another vicariance event accounts for the divergence of *H. sistanensis* from the common ancestor of *H. juliae* and *H. navidpouri* (node 54; relative probability: 1.00).

According to the BBM analysis, four dispersal events and four vicariance events occurred among the species of *Hottentotta*. Dispersal and vicariance events occurred in the northern and southern Zagros Mountains (node 67; relative probability: 0.46), separating Clade A from Clade B, and in the southern Zagros Mountains and the Makran region, separating *H. sistanensis* from the common ancestor of *H. juliae* and *H. navidpouri* (node 54; relative probability: 0.81).

**Figure 5:**
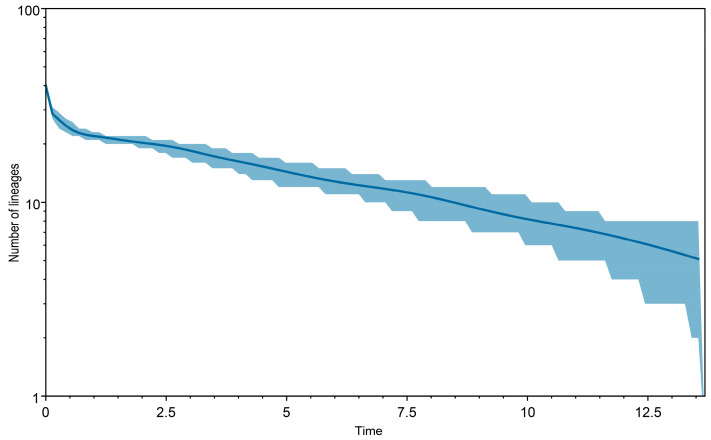
Lineage through time plot of *Hottentotta* Birula, 1908. Horizontal axis (time) indicates millions of years whereas vertical axis indicates log-transformed number of lineages; blue line illustrates diversification of *Hottentotta* based on multilocus species tree whereas blue shaded area represents 95% confidence interval, indicating uncertainty in diversification dynamics through time.

According to the S-DIVA and BBM analyses, 11 and 13 divergence events occurred within the northern and southern Zagros Mountains, respectively, whereas one and two divergence events occurred in the Makran region and the Arabian Peninsula, respectively, revealing the role of allopatric speciation in the diversification of *Hottentotta* in the Zagros Mountains (Figure 5). Six divergence events are evident in northern Africa.

## 4. Discussion

This study presents the first molecular phylogeny and historical biogeographical analysis of *Hottentotta* in the Iranian Plateau and Zagros Mountains. The high degree of genetic diversity among species of the genus underscores the role of the Iranian Plateau and the Zagros Mountains in driving their diversification.

### 4.1. Hottentotta Systematics

The phylogenetic analyses presented here confirmed the monophyly of *Hottentotta* and revealed a distinct clade of species inhabiting the Iranian Plateau and the Zagros Mountains (Figure 2 and Figure 3). Although the Iranian species formed a well-supported clade, the African species were paraphyletic with respect to the Asian species.

The phylogeny revealed several convergent patterns of coloration (Figure 2). For example, yellowish base coloration with infuscate pedipalps, partially infuscate tergites and/or infuscate posterior metasomal segments and telson evolved in multiple species, as did completely infuscate carapace, pedipalps, tergites, metasoma and telson, with or without infuscate legs. Morphologically similar species, like *H. gentili*, *H. schach* and *H. zagrosensis*, were not found to be closely related. This was also the case with species previously assumed to be closely related. For example, according to Kovařík [53], *H. navidpouri* was described based on specimens originally identified as *H. saulcyi* and considered closely related to it, whereas *H. juliae* was described based on the specimens originally identified as *H. schach*. However, the phylogeny did not reveal close relationships between *H. navidpouri* and *H. saulcyi* or between *H. juliae* and *H. schach*.

Although the phylogeny confirmed the monophyly of all species of *Hottentotta* included in the analysis, the taxonomic validity and phylogenetic relationships of several other species from northeast Africa, Iran, Afghanistan, Pakistan and India remain unresolved.

### 4.2. Palaeobiogeography of Hottentotta and Divergence Time Estimation

Divergence time estimation and historical biogeographical analyses (S-DIVA and BBM) indicate that the common ancestor of *H. hottentotta*, *H. franzwerneri* and *H. gentili* diverged from the common ancestor of *H. jayakari*, *H. minax* and the Iranian clade during the Early Miocene (17.56 Ma, 95% HPD: 13.68–22.31; Burdigalian age). These findings support the hypothesis that the Iranian species of *Hottentotta* originated from an African ancestor and subsequently dispersed to their current ranges.

**Figure 6:**
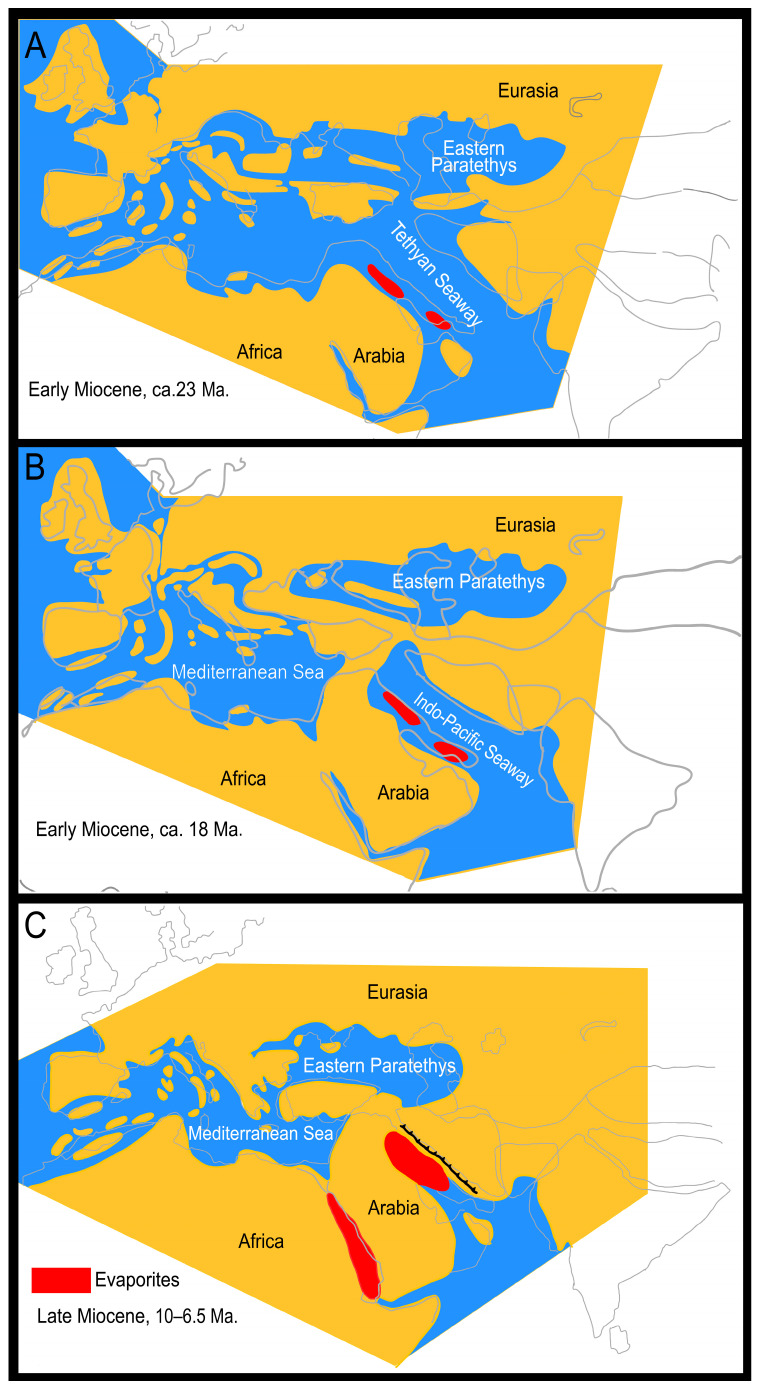
Palaeogeography of Eurasia and Africa at 33.9–27.8 Ma (**A**), 20.4–16 Ma (**B**), and 11.6–7.2 Ma (**C**), after [56].

The present distributions of Afro-Arabian and Iranian *Hottentotta* are most plausibly attributed to major geomorphological processes and plate tectonic events during the Cenozoic. At the Oligocene/Miocene boundary, a deep trough between the Arabian and Iranian plates, known as the Tethyan Seaway (Figure 6A), connected the Mediterranean Sea with the Atlantic and the Indo-Pacific Oceans [54,55]. Closure of the Tethyan Seaway occurred gradually, starting at ca. 19.0 Ma, and continuing until ca. 18 Ma [56,57]. Fossil evidence from terrestrial mammals indicates that prior to its final closure (before 18 Ma), transient or intermittent land connections between Africa and Eurasia allowed periodic episodes of faunal exchange [58]. This faunal exchange was facilitated by the collision of the Afro-Arabian and Eurasian plates, which resulted in the formation of the *Gomphotherium* Landbridge, an emergent corridor that temporarily linked both landmasses [23,56,58,59]. Prior to this tectonic event, faunal exchange between Eurasia, the Arabian Plate, and thus Africa was substantially restricted during the Early Miocene, as the open Tethyan Seaway provided an effective geographical barrier to terrestrial animal dispersal.

The continental rifting between the African and Arabian plates initiated during the Oligocene–Miocene transition, approximately 30–25 Ma, was marked by the opening of the Red Sea Rift. This tectonic event established the Red Sea as an agent of vicariance, progressively fragmenting previously contiguous populations of terrestrial taxa. The ensuing geographical isolation facilitated subsequent divergence and radiation within the Arabian Peninsula. This is supported by molecular phylogenetic analyses of the gecko genus *Hemidactylus* Oken, 1817, which attributed a major divergence to this vicariance event [60]. Divergence time estimation and historical biogeographical analyses indicate that the separation of *H. jayakari* from its African congeners occurred approximately 22–15 Ma, a timeframe that aligns with the period of active tectonic fragmentation between the African and Arabian landmasses.

The Iranian species of *Hottentotta* began diverging during the Early Miocene (14.78 Ma 95% HPD: 11.82–17.72; Langhian age) probably due to seismic activity, volcanism, orogeny and associated palaeoclimatic changes driven by tectonic collision of the Arabian and Eurasian plates and associated basin dynamics [61,62,63,64,65] (Figure 6B). According to the time-calibrated tree, further divergence among lineages which colonized the northern and southern Zagros Mountains, and the central Iranian Plateau, continued from the Middle to Late Miocene (approximately 11.63–5.33 Ma) (Figure 6C). Consequently, climatic and geomorphological changes during the Miocene epoch, especially in the Zagros Mountains, represent a crucial period in the diversification of *Hottentotta*.

Taken together, the S-DIVA, BBM, and BioGeoBEARS model test results indicate that the biogeographical history of *Hottentotta* was shaped primarily by long-term regional persistence, geographical subdivision, and rare but consequential dispersal events. Both S-DIVA and BBM identified the northern and southern Zagros regions as major centers of in situ diversification, with particularly high levels of within-area divergence in the southern Zagros, which emerges as an important source area for dispersal to adjacent regions, including the Makran and Africa. The Makran appears largely as a recipient region with limited internal diversification, whereas the Arabian Peninsula shows comparatively fewer inferred dispersal connections. At a broader scale, both S-DIVA and BBM inferred similar numbers of dispersal and vicariance events and detected no extinction signal, suggesting that diversification was driven mainly by geographical isolation rather than widespread range loss. These patterns are consistent with the complex tectonic history of the region, particularly the uplift and segmentation of the Zagros Mountains and the progressive reorganization of land connections among Iran, Arabia, and Africa, which likely promoted isolation while intermittently permitting faunal exchange [57,58,59]. Consistent with this scenario, BioGeoBEARS model testing favored models incorporating founder-event speciation (+*j*). The near-zero estimates of anagenetic dispersal indicate that gradual range expansion was rare, and that colonization/recolonization of new regions probably occurred through infrequent jump dispersal events followed by speciation. This hypothesis is consistent with the sedentary ecology and low vagility of scorpions [47], in which rare founder events, against a backdrop of tectonically driven vicariance and long-term regional persistence, can have a disproportionate influence on lineage diversification.

### 4.3. Miocene Climate Change in the Zagros Mountains

During the Miocene epoch, the Zagros region experienced major climatic transitions driven by tectonic uplift, regional orogeny, and evolving atmospheric circulation [66]. The Early to Middle Miocene was marked by warm, humid conditions, fostering the development of extensive shallow marine carbonate platforms such as the Asmari Formation [67]. These relatively stable, subtropical climatic conditions supported high biodiversity. By the Middle to Late Miocene, however, the climate in the Zagros began shifting toward increased aridity [68]. This change is evident in the transition from carbonate-dominated to clastic-dominated sequences such as the Agha Jari Formation, indicating greater terrestrial influence and reduced precipitation [69]. The orographic effect of the uplifting Zagros Mountains also contributed to regional drying by blocking moist air masses, creating rain shadows and promoting the spread of savanna-like vegetation [70]. The progressive aridification altered vegetation cover and hydrology, influencing faunal distributions and shaping evolutionary pathways [70]. The drying climate likely contributed to faunal turnover and ecological isolation, setting the stage for species diversification among terrestrial taxa like scorpions.

### 4.4. Miocene Chronostratigraphy of the Zagros Mountains

The chronostratigraphy of the Zagros region during the Miocene epoch is defined by a transition from marine to continental depositional systems, marked in particular by the Asmari and Agha Jari formations [71]. The Early Miocene Asmari Formation predominantly comprises shallow marine carbonates, whereas the Middle to Late Miocene Agha Jari Formation reflects fluvial and deltaic sedimentation [72]. This sedimentary evolution introduced a mosaic of environments, including marine basins, coastal plains, and inland deltas, which created natural geographical barriers to the dispersal of terrestrial animals like scorpions. For example, alternating marine transgressions and clastic deltaic environments would have fragmented habitats and restricted gene flow between populations [73]. Allopatric speciation likely occurred as isolated populations adapted to localized ecological conditions [74].

The progressive rise of the Zagros thrust belt provided a further topographical barrier dividing eastern and western biotas [75]. The barrier created by these orogenic and sedimentary changes, combined with climatic fluctuations, contributed to faunal turnover and the emergence of endemic lineages [76]. Thus, Miocene sedimentary environments in the Zagros not only recorded tectono-stratigraphic transitions but actively influenced arthropod diversification through ecological isolation. These climatic changes, together with the accelerating collision of the Arabian plate with the Eurasian landmass during the Pliocene [77], contributed to the formation of unique environmental conditions on the elevated Iranian Plateau.

Recent studies confirm that the Zagros Mountains provided a significant geographical barrier promoting scorpion diversification. Genetic and ecological data reveal that scorpion taxa such as *Hottentotta*, *Mesobuthus* Vachon, 1950, and *Odontobuthus* Vachon, 1950 exhibit deep genetic divergences across this mountain range, consistent with vicariance and allopatric speciation [12,13,18,39]. The orogeny of the Zagros Mountains disrupted gene flow and created distinct ecological niches, leading to cryptic species formation and localized endemism [12,13,39,78]. These findings underscore the role of the Zagros Mountains in shaping arthropod diversity through habitat fragmentation across the arid and semi-arid landscape of the Iranian Plateau in a manner similar to the diversification of vertebrates such as reptiles and amphibians inhabiting the same region [14,15,22,79].

## 5. Conclusions

A historical biogeographical reconstruction based on the phylogeny confirmed the hypothesis that the Zagros Mountains provided a barrier to scorpion dispersal and promoted diversification on the Iranian Plateau. Divergence of the major clades of *Hottentotta* during the Miocene epoch was driven by orogeny of the Zagros Mountains and subsequent paleoclimatic change. Analyses based on a more comprehensive taxon sample, including additional species and populations, will further elucidate the evolutionary history of *Hottentotta*.

## Figures and Tables

**Table 1:** Genbank accession codes of DNA sequences of nuclear 28S rDNA (28S) locus and mitochondrial 12S rDNA (12S), 16S rDNA (16S) and Cytochrome *c* Oxidase Subunit I (COI) loci for species of *Hottentotta* Birula, 1908 and outgroups, with provenance data for vouchers and tissue samples from which DNA was extracted, deposited in the Zoological Museum of Ferdowsi University of Mashhad (ZMFUM), Iran, and the Ambrose Monell Cryocollection (AMCC) at the American Museum of Natural History (AMNH), New York, U.S.A. Abbreviations: *Acras*: *Androctonus crassicauda* (Olivier, 1807); *Meup*: *Mesobuthus eupeus* (C.L. Koch, 1839); *Hfra*, *Hottentotta franzwerneri* (Birula, 1914); *Hgen*, *Hottentotta gentili* (Pallary, 1924); *Hhat*, *Hottentotta hatamtiorum* Amiri et al., 2024; *Hhot*: *Hottentotta hottentotta* (Fabricius, 1787); *Hjay*, *Hottentotta jayakari* (Pocock, 1895); *Hjul*, *Hottentotta juliae* Kovařík et al., 2019; *Hkho*, *Hottentotta khoozestanus* Navidpour et al., 2008; *Hmin*, *Hottentotta minax* (L. Koch, 1875)*; Hnav*, *Hottentotta navidpouri* Kovařík et al., 2018; *Hsau*, *Hottentotta saulcyi* (Simon, 1880); *Hsch*, *Hottentotta schach* (Birula, 1905); *Hsis*, *Hottentotta sistanensis* Kovařík et al., 2018; *Hzag*, *Hottentotta zagrosensis* Kovařík, 1997.

	Voucher	Tissue	Location	Georeference	28S	12S	16S	COI
***Acras***	–	–	IRAQ	–	–	–	–	PQ790619
***Meup***	–	–	IRAN: Mazandaran Province	36.24° N 53.54° E	–	–	–	HM567348
***Hfra***	–	–	MOROCCO: Figuig outskirts	32.08° N 01.24° W	–	–	–	JF820095
	–	–	MOROCCO: Figuig outskirts	32.08° N 01.24° W	–	–	–	JF820094
***Hgen***	–	–	MOROCCO: Oulad Aissa	30.56° N 08.61° W	–	–	–	JF820088
	–	–	MOROCCO: Oulad Aissa	30.56° N 08.61° W	–	–	–	JF820093
***Hhat***	ZMFUM 2069	AMCC [LP 17146]	IRAN: *Khuzestan*: Andimeshk	32.56° N 48.41° E	PP133567	PP133541	PP133556	PP133856
	ZMFUM 1978	AMCC [LP 17133]	IRAN: *Ilam*: Darehshahr	33.10° N 47.48° E	PP133566	PP133534	PP133553	PP135557
	AMNH	AMCC [LP 11063]	IRAN: *Ilam*: Mormori	32.77° N 47.66° E	PP133569	PP133543	PP133555	PP133858
	AMNH	AMCC [LP 4344]	IRAN: *Khuzestan*: Izeh	31.82° N 49.83° E	PP133570	PP133542	PP133554	PP133857
	ZMFUM 2045	AMCC [LP 17141]	IRAN: *Khuzestan*: Masjedsoleiman	32.10° N 49.56° E	PP133568	PP133538	PP133557	PP135558
***Hhot***	–	–	–	–	–	–	–	ON255608
	–	–	–	–	–	–	–	MN106047
***Hjay***	ZMFUM 1972	AMCC [LP 17130]	IRAN: *Hormozgan*: Qeshm	26.44° N 55.55° E	PZ035518	PZ035501	PZ035536	PZ037917
	ZMFUM 1973	AMCC [LP 17131]	IRAN: *Hormozgan*: Qeshm	26.44° N 55.55° E	PZ035519	PZ035502	PZ035537	PZ037918
	AMNH	AMCC [LP 14318]	OMAN: Bani Khazir	23.19° N 57.35° E	PZ035520	PZ035503	PZ035538	PZ037919
***Hjul***	ZMFUM 1955	AMCC [LP 17126]	IRAN: *Fars*: Abadeh	31.10° N 52.40° E	PZ035513	PZ035496	PZ035531	PZ037912
	ZMFUM 1957	AMCC [LP 17127]	IRAN: *Fars*: Eghlid	30.51° N 52.41° E	PZ035514	PZ035497	PZ035532	PZ037913
	ZMFUM 1959	AMCC [LP 17128]	IRAN: *Fars*: Eghlid	30.51° N 52.41° E	PZ035515	PZ035498	PZ035533	PZ037914
***Hkho***	ZMFUM 2067	AMCC [LP 17144]	IRAN: *Khuzestan*: Ahwaz	31.37° N 48.77° E	PZ035528	–	–	PZ037927
	AMNH	AMCC [LP 9996]	IRAN: *Khuzestan*: Rigsefid	31.74° N 49.19° E	PZ035527	PZ035510	PZ035545	PZ037926
***Hmin***	–	–	SUDAN: Gezira	14.50° N 33.50° E	–	–	–	KX648427
	–	–	SUDAN: Gezira	14.50° N 33.50° E	–	–	–	KX648426
	–	–	SUDAN: Gebel Aulia	15.24° N 32.50° E	–	–	–	KX648425
***Hnav***	ZMFUM 1967	AMCC [LP 17129]	IRAN: *Hormozgan*: Bandar Abbas	27.25° N 56.10° E	PZ035516	PZ035499	PZ035534	PZ037915
	ZMFUM 1996	AMCC [LP 17137]	IRAN: *Hormozgan*: Bandar Abbas	27.16° N 56.20° E	PZ035517	PZ035500	PZ035535	PZ037916
***Hsau***	ZMFUM 1976	AMCC [LP 17132]	IRAN: *Lorestan*: Aleshtar	33.98° N 48.33° E	PP133559	PP133533	PP133546	PP133850
	ZMFUM 2046	AMCC [LP 17142]	IRAN: *Lorestan*: Aleshtar	33.88° N 48.28° E	PP133563	PP133539	PP133550	PP133854
	ZMFUM 2063	AMCC [LP 17143]	IRAN: *Lorestan*: Aleshtar	33.96° N 48.33° E	PP133564	PP133540	PP133551	PP133855
	ZMFUM 1999	AMCC [LP 17138]	IRAN: *Lorestan*: Borojerd	33.89° N 48.57° E	PP133560	PP133535	PP133547	PP133851
	ZMFUM 2000	AMCC [LP 17139]	IRAN: *Lorestan*: Borojerd	33.89° N 48.57° E	PP133561	PP133536	PP133548	PP133852
	ZMFUM 2003	AMCC [LP 17140]	IRAN: *Lorestan*: Borojerd	33.89° N 48.57° E	PP133562	PP133537	PP133549	PP133853
	AMNH	AMCC [LP 16871]	IRAQ: Erbil: Qatawi	36.13° N 43.95° E	PP133565	PP133544	PP133552	PP133859
***Hsch***	ZMFUM 1989	AMCC [LP 17134]	IRAN: *Chaharmahal va Bakhtiari*: Ardal	31.99° N 50.65° E	PP133558	PP133532	PP133545	PP133849
	ZMFUM 1991	AMCC [LP 17135]	IRAN: *Chaharmahal va Bakhtiari*: Bazoft	32.09° N 50.01° E	PZ035522	PZ035505	PZ035540	PZ037921
	ZMFUM 2070	AMCC [LP 17147]	IRAN: *Chaharmahal va Bakhtiari*: Bazoft	32.13° N 49.59° E	PZ035521	PZ035504	PZ035539	PZ037920
***Hsis***	ZMFUM 1946	AMCC [LP 17124]	IRAN: *Sistan va Baluchistan*: Saravan	27.21° N 62.21° E	PZ035511	PZ035494	PZ035529	PZ037910
	ZMFUM 1949	AMCC [LP 17125]	IRAN: *Sistan va Baluchistan*: Saravan	27.21° N 62.21° E	PZ035512	PZ035495	PZ035530	PZ037911
***Hzag***	ZMFUM 1993	AMCC [LP 17136]	IRAN: *Kohgiluyeh va Boyer-Ahmad*: Yasuj	30.42° N 51.38° E	PZ035523	PZ035506	PZ035541	PZ037922
	ZMFUM 2068	AMCC [LP 17145]	IRAN: *Kohgiluyeh va Boyer-Ahmad*: Yasuj	30.56° N 50.54° E	PZ035526	PZ035509	PZ035544	PZ037925
	AMNH	AMCC [LP 4345]	IRAN: *Khuzestan*: Izeh	31.82° N 49.83° E	PZ035525	PZ035508	PZ035543	PZ037924
	AMNH	AMCC [LP 9701]	IRAN: *Khuzestan*: Dehdez	31.70° N 50.28° E	PZ035524	PZ035507	PZ035542	PZ037923

**Table 2:** Average pairwise Kimura 2-parameter distances for DNA sequences of nuclear 28S rDNA (28S) locus and mitochondrial 12S rDNA (12S), 16S rDNA (16S) and Cytochrome *c* Oxidase Subunit I (COI) loci among and within (boldface) of *Hottentotta* Birula, 1908. Abbreviations: *Hfra*, *Hottentotta franzwerneri* (Birula, 1914); *Hgen*, *Hottentotta gentili* (Pallary, 1924); *Hhat*, *Hottentotta hatamtiorum* Amiri et al., 2024; *Hhot*, *Hottentotta Hottentotta* (Fabricius, 1787); *Hjay*, *Hottentotta jayakari* (Pocock, 1895); *Hjul*, *Hottentotta juliae* Kovařík et al., 2019; *Hkho*, *Hottentotta khoozestanus* Navidpour et al., 2008; *Hmin*, *Hottentotta minax* (L. Koch, 1875)*; Hnav*, *Hottentotta navidpouri* Kovařík et al., 2018; *Hsau*, *Hottentotta saulcyi* (Simon, 1880); *Hsch*, *Hottentotta schach* (Birula, 1905); *Hsis*, *Hottentotta sistanensis* Kovařík et al., 2018; *Hzag*, *Hottentotta zagrosensis* Kovařík, 1997. Standard error estimate(s) are shown above the diagonal and were obtained by a bootstrap procedure (500 replicates) in MEGA X.

		*Hmin*	*Hsis*	*Hjul*	*Hnav*	*Hjay*	*Hsch*	*Hzag*	*Hsau*	*Hkho*	*Hhat*	*Hfra*	*Hgen*	*Hhot*
**COI**	***Hmin***	**0.01**	0.02	0.02	0.02	0.02	0.02	0.02	0.02	0.02	0.02	0.02	0.02	0.02
	***Hsis***	0.17	**0.00**	0.02	0.02	0.02	0.02	0.02	0.02	0.02	0.02	0.02	0.02	0.02
	***Hjul***	0.15	0.12	**0.00**	0.01	0.02	0.02	0.02	0.02	0.02	0.02	0.02	0.02	0.02
	***Hnav***	0.16	0.12	0.05	**0.01**	0.02	0.02	0.02	0.02	0.02	0.02	0.02	0.02	0.02
	***Hjay***	0.16	0.16	0.16	0.16	**0.02**	0.02	0.02	0.02	0.02	0.01	0.02	0.02	0.02
	***Hsch***	0.13	0.14	0.12	0.12	0.15	**0.05**	0.01	0.02	0.01	0.01	0.02	0.02	0.02
	***Hzag***	0.14	0.13	0.13	0.13	0.13	0.10	**0.04**	0.01	0.01	0.01	0.02	0.02	0.01
	***Hsau***	0.15	0.14	0.12	0.14	0.15	0.12	0.12	**0.02**	0.01	0.01	0.02	0.02	0.02
	***Hkho***	0.14	0.14	0.12	0.12	0.16	0.11	0.10	0.11	**0.08**	0.01	0.02	0.02	0.01
	***Hhat***	0.12	0.13	0.14	0.14	0.13	0.13	0.11	0.12	0.12	**0.07**	0.02	0.02	0.01
	***Hfra***	0.16	0.17	0.16	0.17	0.15	0.16	0.14	0.15	0.14	0.14	**0.00**	0.01	0.02
	***Hgen***	0.13	0.17	0.15	0.15	0.15	0.15	0.14	0.17	0.14	0.14	0.05	**0.01**	0.02
	***Hhot***	0.13	0.14	0.12	0.12	0.12	0.13	0.12	0.14	0.12	0.11	0.11	0.11	**0.02**
**16S**	***Hsis***	–	**0.00**	0.02	0.02	0.03	0.02	0.02	0.02	0.02	0.02	–	–	
	***Hjul***	–	0.08	**0.00**	0.01	0.03	0.02	0.02	0.02	0.02	0.02	–	–	
	***Hnav***	–	0.08	0.04	**0.01**	0.03	0.02	0.02	0.02	0.02	0.02	–	–	
	***Hjay***	–	0.21	0.25	0.25	**0.01**	0.03	0.03	0.03	0.03	0.02	–	–	
	***Hsch***	–	0.12	0.14	0.14	0.24	**0.05**	0.01	0.02	0.02	0.02	–	–	
	***Hzag***	–	0.12	0.13	0.12	0.24	0.10	**0.04**	0.02	0.02	0.02	–	–	
	***Hsau***	–	0.14	0.15	0.15	0.22	0.15	0.17	**0.01**	0.02	0.02	–	–	
	***Hkho***	–	0.12	0.14	0.12	0.22	0.10	0.13	0.15	–	0.02	–	–	
	***Hhat***	–	0.13	0.13	0.14	0.20	0.16	0.15	0.13	0.16	**0.05**	–	–	
**12S**	***Hsis***	–	**0.00**	0.03	0.02	0.04	0.02	0.03	0.03	0.03	0.02	–	–	
	***Hjul***	–	0.13	**0.00**	0.01	0.04	0.03	0.03	0.03	0.03	0.03	–	–	
	***Hnav***	–	0.12	0.04	**0.01**	0.04	0.03	0.03	0.03	0.03	0.03	–	–	
	***Hjay***	–	0.27	0.26	0.26	**0.02**	0.03	0.04	0.04	0.04	0.03	–	–	
	***Hsch***	–	0.15	0.15	0.15	0.24	**0.05**	0.02	0.03	0.03	0.03	–	–	
	***Hzag***	–	0.15	0.16	0.17	0.23	0.14	**0.03**	0.03	0.03	0.03	–	–	
	***Hsau***	–	0.15	0.19	0.21	0.25	0.19	0.18	**0.01**	0.03	0.02	–	–	
	***Hkho***	–	0.17	0.17	0.18	0.28	0.18	0.15	0.22	–	0.03	–	–	
	***Hhat***	–	0.15	0.19	0.20	0.22	0.19	0.17	0.11	0.21	**0.09**	–	–	
**28S**	***Hsis***	–	**0.000**	0.000	0.000	0.003	0.002	0.002	0.004	0.001	0.003	–	–	
	***Hjul***	–	0.000	**0.000**	0.000	0.003	0.002	0.002	0.004	0.001	0.003	–	–	
	***Hnav***	–	0.000	0.000	**0.000**	0.003	0.002	0.002	0.004	0.001	0.003	–	–	
	***Hjay***	–	0.004	0.004	0.004	**0.000**	0.003	0.004	0.004	0.003	0.003	–	–	
	***Hsch***	–	0.002	0.002	0.002	0.006	**0.000**	0.001	0.003	0.001	0.002	–	–	
	***Hzag***	–	0.002	0.002	0.002	0.006	0.000	**0.001**	0.003	0.001	0.002	–	–	
	***Hsau***	–	0.008	0.008	0.008	0.008	0.006	0.006	**0.000**	0.004	0.003	–	–	
	***Hkho***	–	0.002	0.002	0.002	0.006	0.002	0.002	0.008	**0.003**	0.002	–	–	
	***Hhat***	–	0.004	0.004	0.004	0.004	0.002	0.002	0.004	0.004	**0.000**	–	–	

**Table 3:** Statistics of ancestral range estimation model testing for *Hottentotta* Birula, 1908, using BioGeoBEARS. d: dispersal rate; e: extinction; *j*: founder-event speciation.

Model	LnL	*n*	d	e	*j*	AICc	AICc_wt
DEC	−23.18	2	0.0070	0.50	0.00	50.68	0.018
DEC + J	−19.06	3	1.0 × 10^−12^	0.50	0.013	44.79	0.34
DIVALIKE	−24.41	2	0.0077	0.86	0.00	53.14	0.0052
DIVALIKE + J	−19.47	3	1.0 × 10^−12^	1.03	0.015	45.60	0.23
BAYAREALIKE	−25.9	2	0.0055	0.31	0.00	56.13	0.0012
BAYAREALIKE + J	−18.87	3	1.0 × 10^−07^	0.44	0.012	44.41	0.41

## Data Availability

The original data presented in the study are openly available in National Center for Biotechnology Information at: https://www.ncbi.nlm.nih.gov/ (10 December 2025).

## References

[1] Li Y.S., Shih K.M., Chang C.T., Chung J.D., Hwang S.-Y. (2019). Testing the effect of mountain ranges as a physical barrier to current gene flow and environmentally dependent adaptive divergence in *Cunninghamia konishii* (Cupressaceae). Front. Genet..

[2] Machado A.P., Clément L., Uva V., Goudet J., Roulin A. (2018). The Rocky Mountains as a dispersal barrier between barn owl (*Tyto alba*) populations in North America. J. Biogeogr..

[3] Nesbø C., Magnhagen C., Jakobsen K. (1998). Genetic differentiation among stationary and anadromous perch (*Perca fluviatilis*) in the Baltic Sea. Hereditas.

[4] Shahzad K., Jia Y., Chen F.L., Zeb U., Li Z.H. (2017). Effects of mountain uplift and climatic oscillations on phylogeography and species divergence in four endangered *Notopterygium* herbs. Front. Plant Sci..

[5] Su H., Qu L., He K., Zhang Z., Wang J., Chen Z., Gu H. (2003). The Great Wall of China: A physical barrier to gene flow?. Heredity.

[6] Davis S., Heywood V., Hamilton A. (1994). Centers of Plant Diversity: A Guide and Strategy for their Conservation. Vol. 1. Europe, Africa, South West Asia and the Middle East.

[7] Firouz E. (2005). The Complete Fauna of Iran.

[8] Frey W., Kürschner H., Probst W. (1999). Flora and vegetation, including plant species and larger vegetation complexes in Persia. Encycl. Iran..

[9] Olson D.M., Dinerstein E., Wikramanayake E.D., Burgess N.D., Powell G.V., Underwood E.C., D’Amico J.A., Itoua I., Strand H.E., Morrison J.C. (2001). Terrestrial Ecoregions of the World: A New Map of Life on Earth: A new global map of terrestrial ecoregions provides an innovative tool for conserving biodiversity. BioScience.

[10] Zehzad B., Kiabi B.H., Madjnoonian H. (2002). The natural areas and landscape of Iran: An overview. Zool. Middle East.

[11] Ahmadzadeh F., Flecks M., Torki F., Boehme W. (2011). A new species of angular-toed gecko, genus *Cyrtopodion* (Squamata: Gekkonidae), from southern Iran. Zootaxa.

[12] Amiri M., Prendini L., Hussen F.S.S., Aliabadian M., Siahsarvie R., Mirshamsi O. (2024). Integrative systematics of the widespread Middle Eastern buthid scorpion, *Hottentotta saulcyi* (Simon, 1880), reveals a new species in Iran. Arthropod Syst. Phylogeny.

[13] Barahoei H., Prendini L., Navidpour S., Tahir H.M., Aliabadian M., Siahsarvie R., Mirshamsi O. (2022). Integrative systematics of the tooth-tailed scorpions, *Odontobuthus* (Buthidae), with descriptions of three new species from the Iranian Plateau. Zool. J. Linn. Soc..

[14] Boroumand H., Saberi-Pirooz R., Bafti S.S., Böhme W., Ahmadzadeh F. (2024). Speciation in the Iranian Plateau: Molecular phylogeny and evolutionary history of the Persian long-tailed desert lizard. Zool. Scr..

[15] Ghaedi Z., Badri S., Saberi-Pirooz R., Vaissi S., Javidkar M., Ahmadzadeh F. (2021). The Zagros Mountains acting as a natural barrier to gene flow in the Middle East: More evidence from the evolutionary history of spiny-tailed lizards (Uromasticinae: *Saara*). Zool. J. Linn. Soc..

[16] Levine L.D. (1973). Geographical Studies in the Neo-Assyrian Zagros—I. Iran.

[17] Macey J.R., Schulte J.A., Ananjeva N.B., Larson A., Rastegar-Pouyani N., Shammakov S.M., Papenfuss T.J. (1998). Phylogenetic relationships among agamid lizards of the *Laudakia caucasia* species group: Testing hypotheses of biogeographic fragmentation and an area cladogram for the Iranian Plateau. Mol. Phylogenet. Evol..

[18] Mirshamsi O., Sari A., Elahi E., Hosseinie S. (2010). Phylogenetic relationships of *Mesobuthus eupeus* (CL Koch, 1839) inferred from COI sequences (Scorpiones: Buthidae). J. Nat. Hist..

[19] Rastegar-Pouyani E., Rastegar-Pouyani N., Noureini-Kazemi S., Joger U., Wink M. (2010). Molecular phylogeny of the *Eremias persica* complex of the Iranian Plateau (Reptilia: Lacertidae), based on mtDNA sequences. Zool. J. Linn. Soc..

[20] Hatzfeld D., Authemayou C., Van der Beek P., Bellier O., Lavé J., Oveisi B., Tatar M., Tavakoli F., Walpersdorf A., Yamini-Fard F., Leturmy P., Robin C. (2010). The kinematics of the Zagros mountains (Iran). Tectonic and Stratigraphic Evolution of Zagros and Makran During the Mesozoic–Cenozoic.

[21] Agard P., Omrani J., Jolivet L., Mouthereau F. (2005). Convergence history across Zagros (Iran): Constraints from collisional and earlier deformation. Int. J. Earth Sci..

[22] Rastegar-Pouyani N., Vences M., Köhler J., Ziegler T., Böhme W. (2006). Systematics of the genus Asaccus (Sauria: Gekkonidae) on the Zagros mountains, Iran. Proceedings of the 13th Congress of the Societas Europaea Herpetologica.

[23] Cain S., Loria S.F., Ben-Shlomo R., Prendini L., Gefen E. (2021). Dated phylogeny and ancestral range estimation of sand scorpions (Buthidae: *Buthacus*) reveal Early Miocene divergence across land bridges connecting Africa and Asia. Mol. Phylogenet. Evol..

[24] Esposito L.A., Prendini L. (2019). Island ancestors and New World biogeography: A case study from the scorpions (Buthidae: Centruroidinae). Sci. Rep..

[25] Gantenbein B., Keightley P.D. (2004). Rates of molecular evolution in nuclear genes of east Mediterranean scorpions. Evolution.

[26] Gantenbein B., Largiadèr C.R. (2003). The phylogeographic importance of the Strait of Gibraltar as a gene flow barrier in terrestrial arthropods: A case study with the scorpion *Buthus occitanus* as model organism. Mol. Phylogenet. Evol..

[27] Loria S.F., Ehrenthal V.L., Nguyen A.D., Prendini L. (2022). Climate relicts: Asian scorpion family Pseudochactidae survived Miocene aridification in caves of the Annamite Mountains. Insect Syst. Divers..

[28] Loria S.F., Prendini L. (2020). Out of India, thrice: Diversification of Asian forest scorpions reveals three colonizations of Southeast Asia. Sci. Rep..

[29] Loria S.F., Prendini L. (2021). Burrowing into the forest: Phylogeny of the Asian forest scorpions (Scorpionidae: Heterometrinae) and the evolution of ecomorphotypes. Cladistics.

[30] Barahoei H., Navidpour S., Aliabadian M., Siahsarvie R., Mirshamsi O. (2020). Scorpions of Iran (Arachnida: Scorpiones): Annotated checklist, DELTA database and identification key. J. Insect Biodivers. Syst..

[31] Mirshamsi O., Sari A., Hosseinie S. (2011). History of study and checklist of the scorpion fauna (Arachnida: Scorpiones) of Iran. Prog. Biol. Sci..

[32] Kovařík F. (2007). A revision of the genus *Hottentotta* Birula, 1908, with descriptions of four new species (Scorpiones, Buthidae). Euscorpius.

[33] Navidpour S., Kovařík F., Soleglad M.E., Fet V. (2008). Scorpions of Iran (Arachnida, Scorpiones). Part I. Khoozestan province. Euscorpius.

[34] Navidpour S., Nayebzadeh H.H., Soleglad M.E., Fet V., Kovařík F., Kayedi M.H. (2010). Scorpions of Iran (Arachnida: Scorpiones). Part VI. Lorestan Province. Euscorpius.

[35] Yağmur E.A., Moradi M., Tabatabaei M., Jafari N. (2022). Contributions to the scorpion fauna of Iran. Part II. *Hottentotta akbarii* sp. nov. from the Fars Province (Scorpiones: Buthidae). Serket.

[36] Hijmans R., Guarino L., Bussink C., Mathur P., Cruz M., Barrentes I., Rojas E. (2004). DIVA-GIS, Version 5.0. A Geographic Information System for the Analysis of Species Distribution Data.

[37] Prendini L., Ehrenthal V.L., Loria S.F. (2021). Systematics of the relictual Asian scorpion family Pseudochactidae Gromov, 1998, with a review of cavernicolous, troglobitic, and troglomorphic scorpions. Bull. Am. Mus. Nat. Hist..

[38] Prendini L., Loria S.F. (2020). Systematic revision of the Asian forest scorpions (Heterometrinae Simon, 1879), revised suprageneric classification of Scorpionidae Latreille, 1802, and revalidation of Rugodentidae Bastawade et al., 2005. Bull. Am. Mus. Nat. Hist..

[39] Azghadi S., Mirshamsi O., Navidpour S., Aliabadian M. (2014). Scorpions of the genus *Odontobuthus* Vachon, 1950 (Scorpiones: Buthidae) from Iran: Phylogenetic relationships inferred from mitochondrial DNA sequence data. Zool. Middle East.

[40] Edgar R.C. (2004). MUSCLE: Multiple sequence alignment with high accuracy and high throughput. Nucleic Acids Res..

[41] Kumar S., Stecher G., Li M., Knyaz C., Tamura K. (2018). MEGA X: Molecular evolutionary genetics analysis across computing platforms. Mol. Biol. Evol..

[42] Akaike H., Petrov B.N., Csáki F. (1973). Information theory as an extension of the maximum likelihood principle. Second International Symposium on Information Theory.

[43] Posada D. (2008). jModelTest: Phylogenetic model averaging. Mol. Biol. Evol..

[44] Huelsenbeck J.P., Ronquist F. (2001). MRBAYES: Bayesian inference of phylogenetic trees. Bioinformatics.

[45] Rambaut A., Drummond A. (2009). TRACER: MCMC Trace Analysis Tool Version v. 1.5.0.

[46] Drummond A.J., Rambaut A. (2007). BEAST: Bayesian evolutionary analysis by sampling trees. BMC Evol. Biol..

[47] Bryson R.W., Savary W.E., Prendini L. (2013). Biogeography of scorpions in the *Pseudouroctonus minimus* complex (Vaejovidae) from south-western North America: Implications of ecological specialization for pre-Quaternary diversification. J. Biogeogr..

[48] Graham M.R., Jaeger J.R., Prendini L., Riddle B.R. (2013). Phylogeography of Beck’s desert scorpion, *Paruroctonus becki*, reveals Pliocene diversification in the eastern California Shear Zone and postglacial expansion in the Great Basin Desert. Mol. Phylogenet. Evol..

[49] Rambaut A., Drummond A.J., Xie D., Baele G., Suchard M.A. (2018). Posterior summarization in Bayesian phylogenetics using Tracer 1.7. Syst. Biol..

[50] Yu Y., Harris A.J., He X. (2010). S-DIVA (Statistical Dispersal-Vicariance Analysis): A tool for inferring biogeographic histories. Mol. Phylogenetics Evol..

[51] Yu Z., Liu L., Xu Y., Wang L., Teng X., Li X., Dai J. (2015). Characterization and biological activities of a novel polysaccharide isolated from raspberry (*Rubus idaeus* L.) fruits. Carbohydr. Polym..

[52] Matzke N.J. (2018). BioGeoBEARS: BioGeography with Bayesian (and Likelihood) Evolutionary Analysis with R Scripts.

[53] Kovařík F., Yağmur E.A., Moradi M. (2018). Two new *Hottentotta* species from Iran, with a review of *Hottentotta saulcyi* (Scorpiones: Buthidae). Euscorpius.

[54] Hempton M.R. (1987). Constraints on Arabian plate motion and extensional history of the Red Sea. Tectonics.

[55] Cavazza W., Cattò S., Zattin M., Okay A.I., Reiners P. (2018). Thermochronology of the Miocene Arabia-Eurasia collision zone of southeastern Turkey. Geosphere.

[56] Rögl F. (1999). Mediterranean and Paratethys. Facts and hypotheses of an Oligocene to Miocene paleogeography (short overview). Geol. Carpathica.

[57] Koufos G.D., Kostopoulos D.S., Vlachou T.D. (2005). Neogene/Quaternary mammalian migrations in eastern Mediterranean. Belg. J. Zool..

[58] Harzhauser M., Kroh A., Mandic O., Piller W.E., Göhlich U., Reuter M., Berning B. (2007). Biogeographic responses to geodynamics: A key study all around the Oligo–Miocene Tethyan Seaway. Zool. Anz..

[59] Hou Z., Li S. (2018). Tethyan changes shaped aquatic diversification. Biol. Rev..

[60] Šmíd J., Carranza S., Kratochvíl L., Gvoždík V., Nasher A., Moravec J. (2013). Out of Arabia: A complex biogeographic history of multiple vicariance and dispersal events in the gecko genus *Hemidactylus* (Reptilia: Gekkonidae). PLoS ONE.

[61] Bohannon R.G., Naeser C.W., Schmidt D.L., Zimmermann R.A. (1989). The timing of uplift, volcanism, and rifting peripheral to the Red Sea: A case for passive rifting?. J. Geophys. Res. Solid Earth.

[62] Bosworth W., Huchon P., McClay K. (2005). The red sea and Gulf of Aden basins. J. Afr. Earth Sci..

[63] Edgell H.S. (2006). Arabian Deserts: Nature, Origin and Evolution.

[64] Girdler R. (1991). The Afro-Arabian rift system—An overview. Tectonophysics.

[65] Kusky T., Robinson C., El-Baz F. (2005). Tertiary–Quaternary faulting and uplift in the northern Oman Hajar Mountains. J. Geol. Soc..

[66] Kaveh-Firouz A., Burg J.-P., Haghipour N., Mandal S.K., Christl M., Mohammadi A. (2023). Tectonics, base-level fluctuations, and climate impact on the Eocene to present-day erosional pattern of the Arabia-Eurasia collision zone (NNW Iranian Plateau and West Alborz Mountains). Tectonics.

[67] Vaziri-Moghaddam H., Seyrafian A., Taheri A., Motiei H. (2010). Oligocene-Miocene ramp system (Asmari Formation) in the NW of the Zagros Basin, Iran: Microfacies, paleoenvironment and depositional sequence. Rev. Mex. Cienc. Geol..

[68] Böhme M., Ilg A., Winklhofer M. (2008). Late Miocene “washhouse” climate in Europe. Earth Planet. Sci. Lett..

[69] Ballato P., Mulch A., Landgraf A., Strecker M.R., Dalconi M.C., Friedrich A., Tabatabaei S.H. (2010). Middle to Late Miocene Middle Eastern climate from stable oxygen and carbon isotope data, southern Alborz mountains, N Iran. Earth Planet. Sci. Lett..

[70] Yousefi M., Mahmoudi A., Vaissi S., Kafash A. (2023). Diversity, diversification and distribution of Iranian vertebrates: The legacy of mountains uplifting, past climatic oscillations, sea level fluctuations and geographical barriers. Biodivers. Conserv..

[71] Allahkarampour Dill M., Vaziri-Moghaddam H., Seyrafian A., Behdad A., Shabafrooz R. (2020). A review of the Oligo–Miocene larger benthic foraminifera in the Zagros basin, Iran; New insights into biozonation and palaeogeographical maps. Rev. Micropaleontol..

[72] Afzal J., Racey A. (2025). Early Miocene larger Foraminifera from Suwadi Island, northern Oman. Geol. Soc. Spec. Publ..

[73] Reichenbacher B., Alimohammadian H., Sabouri J., Haghfarshi E., Faridi M., Abbasi S., Matzke-Karasz R., Fellin M.G., Carnevale G., Schiller W. (2011). Late Miocene stratigraphy, palaeoecology and palaeogeography of the Tabriz Basin (NW Iran, eastern Paratethys). Palaeogeogr. Palaeoclimatol. Palaeoecol..

[74] Noori S., Zahiri R., Yusefi G.H., Rajabizadeh M., Hawlitschek O., Rakhshani E., Husemann M., Rajaei H. (2024). Patterns of zoological diversity in Iran—A review. Diversity.

[75] Yasuhara M., Huang H.-H.M., Reuter M., Tian S.Y., Cybulski J.D., O’dea A., Mamo B.L., Cotton L.J., Di Martino E., Feng R., Hawkins S.J., Lemasson A.J., Allcock A.L., Bates A.E., Byrne M., Evans A.J., Firth L.B., Lucas C.H., Marzinelli E.M., Mumby P.J. (2022). Hotspots of Cenozoic tropical marine biodiversity. Oceanography and Marine Biology: An Annual Review, Volume 60.

[76] Kaya F. (2017). Paleobiogeographic and Paleoecologic Development of the Old-World Savanna Paleobiome During the Neogene. Ph.D. Dissertation.

[77] Cromie C., Scarselli N., Craig J., Khan M.R., Hussain A. (2022). Tectonostratigraphic evolution and hydrocarbon prospectivity south of Gwadar Bay, Makran accretionary wedge, offshore SW Pakistan. J. Pet. Geol..

[78] Mirshamsi O., Azghadi S., Navidpour S., Aliabadian M., Kovařík F. (2013). *Odontobuthus tirgari* sp. nov. (Scorpiones, Buthidae) from the eastern region of the Iranian Plateau. Zootaxa.

[79] Wischuf T., Fritz U. (1996). Eine neue Unterart der Bachschildkröte (*Mauremys caspica ventrimaculata* subsp. nov.) aus dem Iranischen Hochland. Salamandra-Bonn.

